# Ketogenic Diets and Cardio-Metabolic Diseases

**DOI:** 10.3389/fendo.2021.753039

**Published:** 2021-11-02

**Authors:** Weiyue Zhang, Xin Guo, Lulu Chen, Ting Chen, Jiayu Yu, Chaodong Wu, Juan Zheng

**Affiliations:** ^1^ Department of Endocrinology, Union Hospital, Tongji Medical College, Huazhong University of Science and Technology, Wuhan, China; ^2^ Hubei Provincial Clinical Research Center for Diabetes and Metabolic Disorders, Wuhan, China; ^3^ Department of Nutrition and Food Hygiene, School of Public Health, Cheeloo College of Medicine, Shandong University, Jinan, China; ^4^ Department of Nutrition, Texas A&M University, College Station, TX, United States

**Keywords:** ketogenic diets, metabolic diseases, obesity, diabetes mellitus, cardiovascular complications

## Abstract

While the prevalence of cardio-metabolic diseases (CMDs) has become a worldwide epidemic, much attention is paid to managing CMDs effectively. A ketogenic diet (KD) constitutes a high-fat and low-carbohydrate diet with appropriate protein content and calories. KD has drawn the interests of clinicians and scientists regarding its application in the management of metabolic diseases and related disorders; thus, the current review aimed to examine the evidences surrounding KD and the CMDs to draw the clinical implications. Overall, KD appears to play a significant role in the therapy of various CMDs, which is manifested by the effects of KDs on cardio-metabolic outcomes. KD therapy is generally promising in obesity, heart failure, and hypertension, though different voices still exist. In diabetes and dyslipidemia, the performance of KD remains controversial. As for cardiovascular complications of metabolic diseases, current evidence suggests that KD is generally protective to obese related cardiovascular disease (CVD), while remaining contradictory to diabetes and other metabolic disorder related CVDs. Various factors might account for the controversies, including genetic background, duration of therapy, food composition, quality, and sources of KDs. Therefore, it’s crucial to perform more rigorous researches to focus on clinical safety and appropriate treatment duration and plan of KDs.

## Introduction

Cardio-metabolic diseases (CMDs) have become a worldwide epidemic, as demonstrated by an increased prevalence of obesity, diabetes mellitus (DM), metabolic syndrome, cardiovascular disease (CVD), and chronic kidney disease (CKD), and culpable to a significant global financial burden (1). CVDs comprise a wide range of diseases detrimental to cardiac and vascular function (2, 3). To decrease cardiovascular (CV) mortality and related economic burden, it’s important to reduce CV risk factors and employ appropriate therapy in developed countries (4). Various established risk factors such as age, gender, genetic heritage, smoking, high blood pressure, poor eating habits, type 2 diabetes mellitus, dyslipidemia and obesity had been accounted for the development and progression of cardiovascular diseases (CVD).

Dietary factors that profoundly influence human health are linked to cardiovascular disease and other chronic metabolic conditions such as obesity and type 2 diabetes (5); thus, dietary interventions have become an essential component in managing cardiovascular risks (6).

A ketogenic diet (KD) is a high-fat, low-carbohydrate diet with appropriate protein content and calories (7). A traditional KD consists of a 4:1 ratio of fats to carbohydrates and protein, with 90% of the calories from fat, 8% from protein, and only 2% from carbohydrate (8). In recent years, to improve compliance and imitate the effects of classic KD, alternative protocols with different formulations of KD have been proposed (9), including 3:1 KD, 2:1 KD, 1:1 KD, the modified Atkins diet (MAD), the medium-chain triglyceride ketogenic diet (MCTKD), the low glycemic index treatment (LGIT) (10, 11) (**Table 1**). With the implementation of KD therapy, a drastic decrease in dietary carbohydrates reduces glucose utilization. In the human body, KD treatment could imitate the metabolic changes of fasting. In addition, some of the beneficial effects of KDs could be attributable to the production of ketones, e.g., β-hydroxybutyrate (BHB), acetoacetate, and acetone in the liver (12).

**Table 1:** Formulations of common ketogenic diets (KDs).

Diet	Percent Total Daily Energy Intake		
	Fat %	Carbohydrate %	Proteins % (g)
Classic KD (4:1 KD)	90	2	8
3:1 KD	87	4	9
2:1 KD	82	8	10
1:1 KD	70	10	20
MAD	60-65	5-10	30
MCTKD	70-75	15-19	10
LGIT	60	10	30

MAD, the modified Atkins diet; MCTKD, the medium chain triglyceride ketogenic diet; LGIT, the low glycemic index treatment.

KD was firstly used as a dietary treatment for epilepsy in the 1920s (8). However, with the progress in antiepileptic drugs (AEDs) development and application, the clinical use of KDs in epilepsy has dramatically decreased. Interestingly, about one-third of patients receiving epilepsy treatment couldn’t gain significant relief from the disease, and the KD regained scientists’ attention and became a choice for application in drug-resistant or difficult-to-treat epilepsies (13, 14). Apart from neurological diseases, KD has recently shown promising efficacy in a wide variety of diseases, including various cancers and metabolic diseases. Ovarian cancer, for instance, may reveal significantly better clinical outcomes under KD intervention, as revealed by a systematic review of randomized controlled trials (15). Furthermore, KD intervention has been found to inhibit tumor progression or mitigate cachexia symptoms (16).

**Figure 1:**
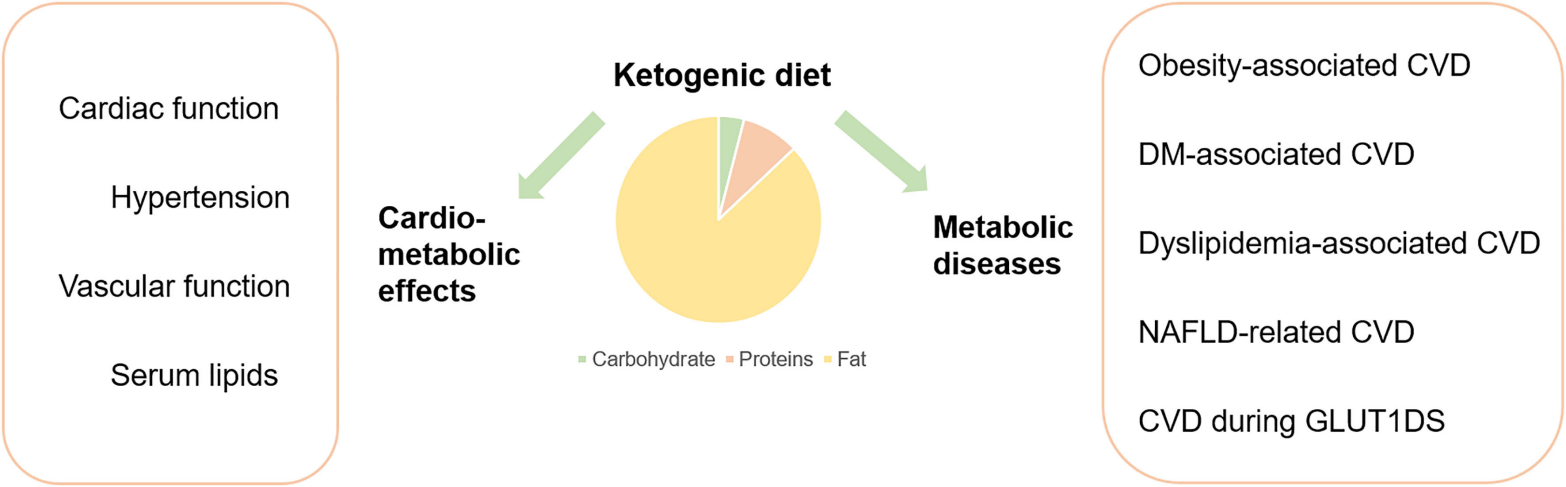
Graphic abstract.

KD has drawn more interest and gradually become an elective dietary intervention choice for CMDs (17). Moreover, it is significantly effective in mitigating various metabolic diseases, including obesity (18, 19), glucose transporter type 1 deficiency syndrome (GLUT1DS) (20), and pyruvate dehydrogenase (PDH) deficiency (21). Meanwhile, due to the uncertainty of dietary interventions, different voices have also occurred regarding the safety issues and drawbacks of employing KD. The clarity on how KD influences cardiovascular and metabolic diseases remains unclear. Therefore, the current review highlighted pertinent information concerning KD and CMDs (**Figure 1**). Qualified studies reflecting the advantages or disadvantages of KD in CMDs were all equally considered and incorporated without bias.

## Effects of Ketogenic Diets on Metabolic Diseases

Various metabolic diseases have been recognized as cardiovascular risk factors, including diabetes mellitus, obesity and other metabolic diseases (22). The disrupted glucose and lipid metabolism lead to abnormal oxidative stress, inflammatory, vasoactive factors, cardiac and vascular function, and finally elevate the risk of CVD (23). KD can regulate metabolic profiles and may consequently regulate the risk of CVD.

As for the management of obesity, most studies indicated that KDs were efficient at weight loss (24, 25), especially in reducing food intake in humans and elevating energy consumption in animals (18, 19). Studies concerning the effects of KDs on body composition changes reported that KD-fed mice had an increased fat mass percentage than regular-chow fed mice (26, 27), and reduced (28) or no differences (26) in the percentage of lean body mass between diets; whereas in humans, weight loss affects both fat and lean mass (29). Obesity is commonly connected with insulin resistance and type 2 diabetes mellitus (T2DM), in which systemic ketone body metabolism is perturbed (30, 31). As such, the weight-loss effect of KDs is expected to be beneficial for diabetes; although it remains disputable that KD induces insulin resistance. There are studies indicating that KD led to ameliorated glucose homeostasis and reduced antidiabetic medications in T2DM subjects (32, 33), even with reduced baseline insulin levels and elevated insulin sensitivity in diabetic rats (34, 35). Moreover, the study by Farrés et al. (33) appeared to offer a plausible explanation of how KDs bring about the anti-diabetic effect, which might be attributable to the anti-inflammatory effect of KD itself and beneficial effects of the altered lipid metabolism on diabetes effector proteins. However, certain studies suggested that KD reduced glucose and insulin levels while inducing insulin resistance and glucose intolerance in rats (27, 36). Besides, utilizing KD in adults with type 1 diabetes mellitus (T1DM) is associated with dyslipidaemia and a high number of hypoglycaemic episodes apart from excellent HbA1c levels and little glycaemic variability (37). Thus, in T1DM, the safety issues are considerable; while in T2DM, more studies are needed to address how KD might impact on insulin resistance and other aspects.

In Polycystic ovarian syndrome (PCOS), KD appears to be a valuable non-pharmacological treatment. A 24-week low-carbohydrate KD (38) and a 12-week ketogenic Mediterranean diet with phyoextracts (39) were both reported to lead to remarkable improvement in body weight, percentages of free testosterone, LH/FSH ratios, and insulin levels in women with PCOS and obesity/overweight. Besides, KD has also been proven effective in other metabolic diseases including glucose transporter type 1 deficiency syndrome (GLUT1DS) (20), pyruvate dehydrogenase (PDH) deficiency (40), phosphofructokinase (PFK) deficiency and glycogenosis type V (McArdle disease) (41).

In summary, KDs are recommended in some inherited metabolic diseases and PCOS, while the effects of KDs on diabetes and some other metabolic diseases remain controversial. Rigorously-designed long-term studies are warranted to evaluate the effects and the safety problems of KDs and further evaluate whether the impact of KDs can be maintained.

## Cardiometabolic Effects of Ketogenic Diets

The occurrence and development of CMDs are closely related to systemic chronic low-grade inflammation characterized by the continuous increase of circulatory inflammatory factors (42). Dietary pattern is one of the important factors that affect chronic inflammatory states (43). Thus, the effects of dietary pattern on CMDs arouse scientists’ interest and a large number of studies have focused on the cardiometabolic effects of KD. Apart from the above-mentioned metabolic diseases, the effects of KD on cardiac function, hypertension, vascular function and lipid profile have also been studied.

### Ketogenic Diets Regulate Cardiac Function

The effects of KD on cardiac health have been widely investigated, but researches concerning the effects of KD on cardiac functions provided a few relatively controversial data. Studies generally suggest that KD intake benefited cardiac metabolic efficiency and acted as a cardioprotective antioxidant. Selvaraj et al. (44) reviewed current evidence surrounding the use of therapeutic ketosis including KD in heart failure (HF) and pointed out its potential benefit in HF, particularly in HF with reduced ejection fraction. Further, Balietti et al. (45) found that an 8-week supplementation of medium-chain triglycerides KD (MCT-KD) to late-adult rats partly restored age-related decrease of succinic dehydrogenase (SDH) activity and metabolically active mitochondria, which might offset senescent alterations leading to apoptosis-induced myocardial atrophy and failure. Another study with a similar conclusion indicated that a 19-week low carbohydrate KD following global ischemic injury significantly increased the numbers of mitochondria in cardiac muscles and the reperfusion recovery of coronary flow (46). As such, the two studies demonstrated that KD was cardio-protective in terms of regulating cardiac energy metabolism including mitochondrial capability. However, some studies suggested that KD might be just not harmful to cardiac functions. A study utilizing KD for at least 12 months on cardiac functions in intractable epilepsy patients suggested that the KD used appeared to have no negative impact on ventricular functions in epileptic children in the midterm (47). Similarly, a 6-month KD therapy didn’t affect electrocardiogram outcomes in the drug-resistant children with epilepsy (48). The subjects in these two studies are both epileptic children, which cannot represent all the patients who might use KD therapy. Thus, we can still stay optimistic about the effects of KD on cardiac functions.

Studies have also been conducted concerning the mechanism of how KD might affect cardiac health. Abnormal substrate metabolism is one of the major changes of insulin resistance and diabetic myocardium (49). Given this, changes in the regulation of myocardial ketone body metabolism appear to be a novel diagnostic biomarker of altered ketolytic capacity. Wentz et al. (50) utilized ketogenic nutritional mouse models (24 h of fasting and a very low carbohydrate ketogenic diet) to demonstrate that cardiac muscle engages a metabolic response that limits ketone body utilization. Specifically, the results revealed that unmetabolized substrate concentrations were higher within the hearts of ketogenic diet-fed mice. Furthermore, a recent study suggested that a KD or a high-fat diet could reverse the structural, metabolic and functional remodeling of non-stressed cMPC1-/- (cardiomyocyte-restricted deletion of subunit 1 of mitochondrial pyruvate carrier) mouse hearts (51). A KD of 3 weeks before transverse aortic constriction was already enough to rescue cMPC1-/- hearts from rapid decompensation and early mortality after pressure overload. Another study also indicated that a high-fat, low-carbohydrate KD could completely reverse progressively developed cardiac dilation and contractile dysfunction in mice with cardiac-specific deletion of Mpc2 (CS-MPC2-/-) (52). Accordingly, KD therapy might be promising in improving cardiac fat metabolism to prevent or reverse cardiac dysfunction and remodeling in MPC deficiency.

**Figure 2:**
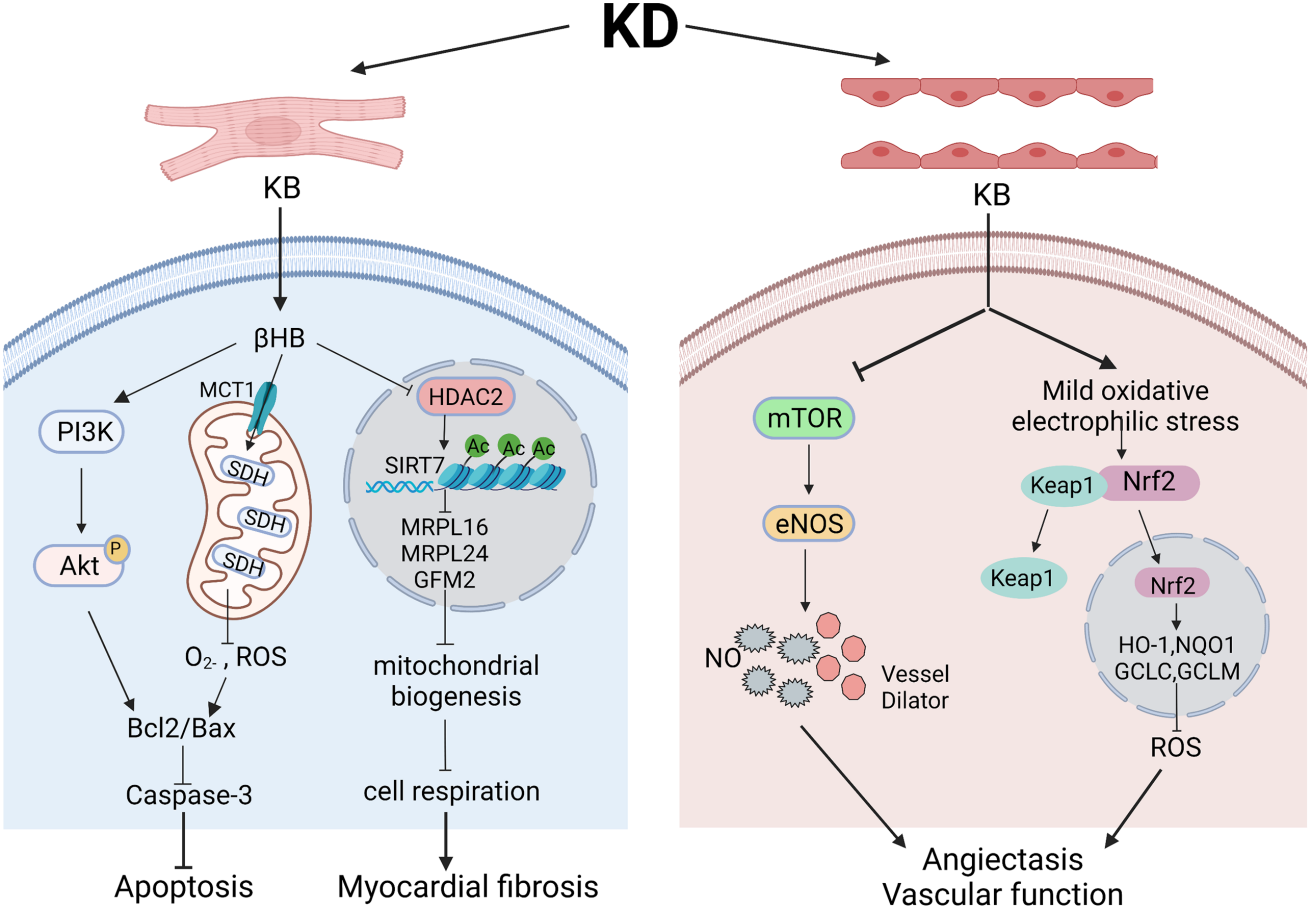
The role and mechanism of ketogenic diets in cardiac function and vascular function. Various pathways might underly the effects of ketogenic diets in cardiomyocytes and endothelial cells in different models. In cardiomyocytes, βHB regulates PI3K/Akt pathway and SDH in mitochondria to finally ameliorate cell apoptosis. However, elevated βHB might also acts through inhibiting HDAC2 and influencing mitochondrial biogenesis, leading to myocardial fibrosis. In endothelial cells, ketone bodies can functions through inhibiting mTOR pathway to regulate the level of eNOS, subsequently dilating blood vessels and enhancing vascular function. Besides, elevated ketone bodies can give rise to mild oxidative/electrophilic stress, activate Nrf2 in cytoplasm and enhance antioxidant gene expression, which lead to lowered ROS level and improved vascular functions. Created with BioRender.com.

As mentioned above, KDs are generally cardioprotective, which might be attributable to the effects of KDs on cardiac metabolism, such as ketone body metabolism and energy metabolism including mitochondrial capability (**Figure 2**). Despite the evidence supporting the cardioprotective effect of KDs, another study utilizing KD on cardiac remodeling in spontaneously hypertensive rats (SHRs) suggested that KD might deteriorate cardiac remodeling in the hypertensive heart and warranted fully evaluation of its reliability before clinical use (53). The different pathogenesis backgrounds of hypertension might account for the different results. More studies with larger samples, longer follow-up duration, and standardized basic health status can be conducted to further clarify the role of KDs in cardiac functions and other potential mechanisms.

### Ketogenic Diets and Hypertension

Attention has also been paid to the effects of KD in hypertension. Most studies showed positive effects of KD in hypertension. Castellana et al. (54) suggested that very-low-calorie ketogenic diet (VLCKD) manifested improvements in hypertension, type 2 diabetes and dyslipidemia, apart from being a promising lifestyle intervention for overweight and obesity. Another study incorporating 377 patients across Italy drew a similar conclusion that VLCKD could significantly lower SBP in three months (55). Even a short-term 4-week KD with micronutrient supplementation could result in improved hypertension control and in a reduction for the usage of hypertension medications in patients with preoperative T2DM and hypertension (56). Increasing ketone bodies by nutritional interventions of ketone bodies or their precursors, such as 1,3-Butanediol, was also reported to attenuate hypertension (57). However, Guo et al. (58) revealed that subjecting spontaneously hypertensive rats (SHRs) to KD for 4 weeks aggravated hypertension, increased the expression of IL1-β and TNF-α, impaired endothelium-dependent relaxation and decreased CD31 and eNOS expression in mesenteric arteries. This finding is opposite to the previous results; thus, it remind us to be cautious in treating hypertension with KD, and perform more studies to explore the effects of KD on hypertension in human studies and animal models.

The Dietary Approaches to Stop Hypertension (DASH) diet is a classic dietary approach that has been endorsed for patients with elevated blood pressure (BP). Besides, the addition of exercise and weight loss to the DASH diet resulted in even larger BP reductions, greater improvements in vascular and reduced left ventricular mass for obese people with elevated BP (59). The main drawback of DASH might be the difficulty in long-term adherence to this diet. Considering the different components of DASH and KD, KD might become another choice for those people who love a high-fat diet, although we suggest cautious application since the antihypertensive efficacy and side effects of KD under the background of hypertension remain unclear. Interestingly, a review suggested that intermittent fasting could lower both systolic and diastolic blood pressure in human studies and animal studies, possibly through reducing oxidative stress, syncing with circadian rhythm, and inducing a ketogenic state (60). The less consumption of fats is assumed as the reason why intermittent fasting appears to be more beneficial than KD in treating hypertension. Thus, the type of fats consumed in KD therapy is crucial to be considered both in treating hypertension and evaluating its effects.

### Ketogenic Diets Regulate Vascular Functions and Vascular Blood Flow

A study by Keogh et al. (61) has indicated that a very-low-carbohydrate, high-saturated-fat weight-loss diet did not impair FMD. How would the actual KD impact on vascular function and vascular blood flow?

In some studies, KD appears to play a protective role in vascular functions. Ischemic tolerance can reduce brain injury and neurological dysfunction after brain ischemia. Additional to the cardiovascular effects such as higher reperfusion recovery of coronary flow, KD also can enhance brain vascular function. As supported by the results from a study upon feeding a KD to young healthy mice, KD intervention enhanced neurovascular function through reducing mTOR protein expression and increasing eNOS levels (62). Yang et al. (63) discovered that feeding mice with KD-fed mice could remarkably decrease infarct volume and elevate regional cerebral blood flow in both ischemic and reperfusion phases. Besides, while investigating the effects of KB level on HMEC-1 endothelial cells, one study indicated that KB activated transcription factor Nrf2 and elevated the expression of cell antioxidant defending genes *via* inducing moderate oxidative stress (64). Thus, the increased KB level by KD might also lead to these protective effects.

However, as for big vessels such as carotid and aortic artery, the effects of KD remain controversial. For instance, after observing the effect of KD on the vascular structure and functions for at least one year, it was found that KD notably elevated the serum levels of lipids but didn’t significantly affect carotid intima-media thickness, aortic and carotid strain, the stiffness index, distensibility, and elastic modulus (65). Another study by Doksoz et al. (66) also demonstrated that a 6-month KD didn’t affect carotid intima-media thickness and elastic properties of the carotid artery and the aorta. In contrast, in the research of Coppola et al. (67), participants prescribed with KD had higher arterial stiffness parameters, including AIx and beta-index and higher serum levels of cholesterol or triglycerides. Another study revealed that a high-fat KD notably elevated atherogenic apolipoprotein B (apoB)-containing lipoproteins and decreased antiatherogenic HDL cholesterol and urged further researches to investigate whether this diet deteriorates endothelial function and facilitates inflammation and formation of atherosclerotic lesions (68). However, a clinical study involving 26 children after one year and 13 children after two years of KD suggested that the initial influences on arterial function observed within the first year of KD-treatment were reversible and were no longer significant after 2 years of the therapy (69). Therefore, the effects of KD on big vessels such as carotid and aortic artery were reversible and were no longer significant after 1-2 years, which might explain the above results.

### Ketogenic Diets Regulate Serum Lipids

Apart from the cardiometabolic effects mentioned above, the impact of KD on serum CVD biomarkers has also been investigated. Research on 20 normal-weight, normolipidemic men indicated that a 6-week KD notably decreased fasting serum triglyceride, postprandial lipemia, and fasting serum insulin concentrations, tended to increase HDL cholesterol, while not affecting fasting serum total and LDL cholesterol and oxidized LDL (70). These results revealed that short-term KD would not deteriorate CVD risk profile and, indeed, appeared to ameliorate lipid disorders that are characteristics of atherogenic dyslipidemia. Another research also indicated that changes in the ratio of protein to carbohydrate toward higher protein proportion could provide beneficial effects on serum lipids apart from lowering body weight (71).

However, Özdemir et al. (65) pointed out that prescribing patients with at least 12 months KD could significantly elevate serum total and LDL cholesterol and triglyceride at a median of 12.6 months while not affecting HDL level. Moreover, another research (72) found that a 6-month KD could notably increase median triglyceride, total cholesterol, LDL, and HDL. They suggested that classic KD was indeed efficient in treating refractory seizures in children but might give rise to hypercholesterolemia and hypertriglyceridemia.

As such, disputable voices concerning the impact of KDs on serum lipids remain to be settled by future work in this field.

## Ketogenic Diets and Cardiovascular Complications of Metabolic Diseases

The potential effects of KDs on the prevention or treatment of cardiovascular risk factors or diseases have been significantly studied over the past decades. Moreover, various animal and human studies have investigated the role of KDs in regulating cardiovascular complications of obesity, insulin resistance and type 2 diabetes, dyslipidemia, NAFLD, and/or GLUT1DS. However, whether and how KDs influence the cardiovascular risk factors or complications in metabolic diseases remains undetermined.

### Obesity-Associated Cardiovascular Disease

Obesity is closely related to CVD, and complications of CVD are often witnessed in obese patients. Cicero et al. (55) evaluated the effect of a very low carbohydrate ketogenic diet (VLCKD) on overweight-related risk factors of CVD such as blood pressure, lipid levels, and glucose metabolism, and the study found that VLCKD intervention for 3 months was generally safe and found effective in inducing weight loss and improved CV risk factors levels.

Another study recruited a hundred obese patients and prescribed them a ketogenic diet for over six months showed significant improvement in patients’ cardiovascular status in addition to weight reduction (73). Moreover, a meta-analysis study by Bueno et al. (74) assessed the long-term effects of VLCKD on body weight and cardiovascular risk factors. The results indicated that under VLCKD, the participants had a significant reduction in body weight, TAG, and diastolic blood pressure, while increased HDL-C and LDL-C levels were observed. Apart from long-term studies, a study by Ministrini et al. (75) that treated obese patients with VLCKD for 25 days also concluded that VLCKD had positive effects on cardiovascular risk factors, and such a beneficial outcome in the short term is remarkable.

Other studies investigated the effects of modified KDs on cardiovascular risks in obese participants. Perez-Guisado et al. (76) carried out a prospective evaluation in 31 obese participants with “Spanish Ketogenic Mediterranean Diet” (incorporating virgin olive oil as a principal source of fat, moderate red wine intake, green vegetables and salads as the primary source of carbohydrates and fish as the main source of protein, SKMD). The SKMD was found safe and effective interventional approach for improving non-atherogenic lipid profiles and lowering blood pressure while lowering body weight. Similarly, Paoli et al. (77) applied another modified KD that incorporated phytoextracts and ingredients imitating the taste of carbohydrates (ketogenic Mediterranean with phytoextracts, KEMEPHY). The study recruited 106 overweight Rome council employees and revealed a remarkable reduction in body weight, BMI, percentage of fat mass, total cholesterol, LDL-C, TAG and blood glucose while displaying a significant increase in HDL-C after the intervention with KEMEPHY. In addition to good compliance, extra beneficial effects on cardiovascular risk markers and waist circumference were also achieved by the KEMEPHY diet.

Since the beneficial effects of KDs on metabolism and cardiovascular risk factors are similar to those seen after n-3 polyunsaturated fatty acids (omega-3) supplementation, Paoli’s team (78) modified the ketogenic Mediterranean diet with phytoextracts after their previous research through combining with omega-3 supplementation. The results suggested that this newly modified diet can further enhance the beneficial effects on cardiovascular risk factors and inflammation in overweight participants.

The influence of a multi-step dietary program including different dietary patterns has also been evaluated. In an open-label study by Castaldo et al. (79), 73 obese patients entered a rehabilitative multi-step dietary program: a 3-week protein-sparing, very low-calorie KD (<500 kcal/day; Oloproteic Diet) and a 6-week hypocaloric (25–30 kcal/kg of ideal body weight/day), low glycemic index, Mediterranean-like diet (hypo-MD). In both phases, improved glucose and lipid metabolism and blood pressure were observed. Based on this, it was concluded that a dietary program consisting of a KD and a subsequent MD could decrease cardiovascular risks efficaciously in obese patients.

### CVD During Diabetes Mellitus

Diabetes mellitus is often associated with obesity (80), and recent estimates showed that 87.5% of T2DM patients are overweight or obese (24). Moreover, obese subjects are prone to developing hypertension, CVD, and strokes, and the risk is even higher if it co-exists with T2DM (81).

In patients with both T2DM and obesity, LC diets not only cause weight loss but also improve postprandial plasma glucose levels, glucose variability, serum triglycerides, and HDL-C levels (82). Similar results were observed by a 2-year randomized clinical trial study (39) that investigated the effect of an LC diet with high unsaturated fat and low saturated fat on glycemic control and CVD risk factors in overweight or obese patients with T2DM. KD is a low-carbohydrate (LC) and high-fat (HF) diet, which sort of belongs to one type of LC diets. However, one of the potential concerns of KDs is postprandial hyperlipidemia, which leads to significant cardiovascular risks (83).

Studies have found that individuals with pre-diabetes or diabetes who received an earlier LCHF diet revealed several beneficial outcomes, including weight loss, improved insulin sensitivity, glucose homeostasis, and lower fasting blood glucose levels. These outcome improvements also decreased the risks of cardiovascular diseases development (38, 84). Mobbs et al. (28) analyzed the evidence concerning the treatment of diabetes and diabetic complications with a KD. They revealed that a classic KD significantly reduced blood glucose in animal models of type 1 and 2 diabetes and reversed diabetic nephropathy without producing significant cardiovascular risks. Moreover, a study on db/db mice revealed that KD ameliorates cardiac dysfunction by inhibiting apoptosis *via* activating the PI3K-Akt pathway in type 2 diabetic mice and suggested KD as a promising lifestyle intervention against diabetic cardiomyopathy (85).

However, there also are studies with conflicting or controversial findings and opinions. Westman et al. (86) stated that LCHF diets gave rise to decreased appetite, thus the improved surrogate markers of cardiovascular disease resulted from weight loss but not from low carbohydrate intake itself. In an animal study, Abdurrachim et al. (87) investigated the effects of long-term KD on cardiac metabolism and diabetic cardiomyopathy status in lean diabetic Goto-Kakizaki (GK) rats. Upon KDs for 62 weeks, diabetic GK rats displayed decreased blood glucose, triglyceride, and insulin levels, revealing increased blood ketone body levels. Additionally, KDs decreased myocardial ketone body and glucose oxidation and induced cardiac hypertrophy. These results suggested that KDs might lead to maladaptive cardiac metabolic modulation and lipotoxicity and deteriorate diabetic cardiomyopathy in GK rats. Given this, the possible role of KDs in cardiovascular risks of DM remains controversial in rodent models and humans, which warrants more studies for elucidation.

### Dyslipidemia-Associated CVD

As for patients with dyslipidemia, Westman et al. (88) investigated the effect of KDs on serum lipoprotein subclasses to address the concern of KDs on cardiovascular risks. The study was a randomized, two-arm clinical trial involving overweight and hyperlipidemic participants motivated to lose weight. After 6 months, the KD group displayed more significant decreases in medium VLDL, small VLDL, and medium LDL, and more significant increases in VLDL particle size, large LDL, and HDL particle size than the control group. Although the KDs did not decrease total LDL cholesterol, they shifted from small, dense LDL to large, buoyant LDL, thus decreasing CVD risks in these participants.

### NAFLD-Related CVD

NAFLD contributes to CVD through various mechanisms. Weight loss has been commonly recommended for treating obesity-associated NAFLD; meanwhile, LCKD benefits weight loss. Recent studies have revealed an association between LC diets and NAFLD in both rodents and humans. In the study by Garbow et al. (28), mice fed a KD for 12 weeks were lean, euglycemic, ketotic, and hypo-insulinemic but were glucose intolerant and with NAFLD. Also, obese subjects on LC diets displayed enhanced weight loss, improved metabolic parameters and decreased intrahepatic triglyceride content. Nevertheless, long-term KDs led to NAFLD and systemic glucose intolerance in mice (89), negatively impacting CVD. As such, current evidence is insufficient to conclude, and more related studies are warranted to explore how KDs might influence NAFLD-related CVD in the long run.

### CVD During GLUT1DS

GLUT1DS is an inherited but treatable disease concerning cerebral energy metabolism (90). KDs are currently a treatment option for GLUT1DS from infancy into adulthood, raising concerns about long-term cardiovascular risks (43, 60, 69). To address this problem, Heussinger et al. (91) performed a 10-year follow-up study on cardiovascular risk of KDs in GLUT1DS and revealed that dyslipidemia caused by KDs might be transient; and carotid intimal wall thickness (CIMT), BMI and blood pressure parameters remained normal after 10 years. Because of this, Heussinger et al. suggested that cardiovascular risks of KDs in some previous studies appeared to be attributable to inadequate follow-up. Also, a period of at least five years appears to be necessary for evaluating the effect of KDs on lipid parameters. Moreover, the authors recommended KDs as a treatment of choice for GLUT1DS. Another study by Alter et al. (90) also characterized the long-term course of GLUT1DS and followed up for an average of 14.2 years (range = 8.9-23.6). The results indicated that earlier introduction of KDs correlated with better long-term outcomes and KDs seemed to be protective of vital organs. However, GLUT1DS is a rare disease, and therefore, the study cohort’s size and external validity are limited. Long-term follow-up studies are warranted to confirm the above findings further.

## Potential Safety Concerns on KD

The majority of the studies had found KD to be beneficial, but some studies had shown concerns regarding heart functions, liver inflammation and so on.

### The Effects of KD on Heart Functions in Rodents

One study found KD treatment ameliorates cardiac dysfunction by inhibiting apoptosis *via* activating the PI3K-Akt pathway in type 2 diabetic mice, suggesting that the KD is a promising lifestyle intervention offering protection against diabetic cardiomyopathy (85). In contrast, a ketogenic diet may lead to adverse effects on the remodeling in the hypertensive heart *via* mechanisms involving increased mTOR signaling, and they underscore the necessity to evaluate its reliability before clinical use (53). Preclinical studies results indicate that KD also has a potential safety concern, although much evidence suggests that KD is a promising approach for managing CVD.

### The Effects of KD on Hepatic Inflammation in Rodents

As a key factor that triggers or exacerbates CVD risk, liver inflammation is a potential safety concern related to KD. In support of this, a study observed that mice fed with KD sustained unimpaired insulin-induced hepatic Akt phosphorylation and whole-body insulin responsiveness but ultimately developed hepatic endoplasmic reticulum stress, steatosis, cellular injury, and macrophage accumulation (28).

### The Effects of KD on Lipid Profile

KD is enriched in lipid contents, and it’s natural to speculate the potential risk of elevated levels of lipids. Apart from studies indicating the beneficial effects of KD, concerns regarding the elevated level of lipids, including serum total and LDL cholesterol and triglyceride, are subjective while prescribing KD (65, 72). It is reported that KDs are likely to deteriorate levels of total, high-density lipoprotein (HDL) and low-density lipoprotein (LDL) cholesterol, and triglycerides (27, 34, 92) in rodents while doing the opposite in humans (39). These contradictory results might be attributable to the different composition of diets since animal researches generally employ diets higher not only in total fat but also in saturated fat (93). Considering this, it is necessary to compare the fat composition, e.g., content of saturated fat versus unsaturated fat in KDs in long-term studies involving both rodents and humans.

As described above, while considering KD as an exciting approach for managing CMDs, it also is important to be cautious about the potential safety concern associated with KD. While future studies are warranted to confirm and elucidate whether and how KD causes potential safety concerns, it would also be important to consider to modifying KD or combining KD with other healthy diets for managing CMDs.

## Discussion

Despite particular safety concerns, the beneficial and advantageous aspects of KD cannot be denied. Because multiple factors are affecting the results, including using different mouse strains, providing KD with different food compositions, short study duration, etc. In the future, studies essentially need to explore the possible factors influencing the responses to KD and improve KD dietary plan for utilizing KD as a dietary therapy to minimize safety concerns.

### Factors Affecting Ketogenic Diets Responses

#### Genetic Control of the Responses to a Ketogenic Diet

Nutrigenetic research suggested that genetic markers critically regulate nutritional interactions that impact body weight and composition, which lays the foundation for personalized nutrition therapy (94, 95).

Barrington et al. (96) observed that mouse genetic backgrounds determined dietary outcomes on CVD risk. Specifically, the study included mice from four inbred strains (A, B6, FVB, and NOD), which accounted for genetic and phenotypic diversity and examined mice’s metabolic responses to four human-comparable mice diets (American, Mediterranean, Japanese and ketogenic diets). The authors revealed that the effects of these diets on metabolic health were indeed dependent on genetic backgrounds. The outcomes of KD on body composition, glucose metabolism and liver health varied markedly among different strains.

The different diet responses could be partly attributable to the genetic background related to varying dietary therapy compliance. Parnell et al. (97) analyzed the interactions between single nucleotide polymorphisms (SNP) in various cardiometabolic pathways and the intake of different nutrients. Their results indicated that gene-environment (GxE) genes had better responses to plasma cholesterol-lowering or regression of atherosclerotic plaques, primarily through high-energy diets and fat intake.

As mentioned above, genetic background plays a vital role in individual responses to KDs and may consequently influence the effects of KDs. It is of great importance to take genetic background into account when initiating KD therapy.

#### Food Composition, Quality and Sources of KDs Influence the Outcome

As KDs are a kind of macronutrient-focused diet, we should fully consider the food composition, quality and sources to avoid potential drawbacks when starting a KD. As indicated in the research by Seidelmann et al. (98), there was a U-shaped association between the percentage of energy consumed from carbohydrates and mortality: 50–55% carbohydrate intake was associated with minimal mortality risk. In comparison, a percentage of <40% or >70% led to greater mortality risk. Besides, different types of dietary fatty acids have different effects on CVD risk and replacing saturated fatty acid (SFA) with unsaturated fats especially polyunsaturated fatty acids can lead to a significant reduction in CVD risk (99). Moreover, diets that favored plant-derived protein and fat intake were associated with lower mortality than animal-derived protein and fat sources. Thus, the nutrient composition, types and sources should be taken into consideration when prescribing a KD therapy; the diversity in these nutrient details could affect the effects of KD and should be relatively standardized to compare the results.

#### Duration of KD Therapy Affects the Responses

Interestingly, increasing the therapeutic duration of KDs appears to reduce some safety-related problems (34, 35). For instance, long-term follow-up research has demonstrated that dyslipidemia caused by KDs is transient. Moreover, over 10 years, KD therapy has ended with normal vascular function as indicated by carotid artery ultrasound (91).

### Modified KD Dietary Plan

Because of the irreconcilable options on the therapeutic use of KD, several studies concerning modified KD and cardiovascular risks have been performed. The “Spanish Ketogenic Mediterranean Diet” carried out by Perez-Guisado et al. (76) and two modified KDs (KEMEPHY (77) and KEMEPHY with omega-3 supplementation (78)) employed by Paoli et al. have all displayed beneficial effects on cardiovascular risk factors. A combined diet consisting of KD and a subsequent Mediterranean-like diet has been proven to decrease cardiovascular risks in patients (79). Therefore, a modified KD or multi-step dietary program including different diet patterns is promising in resolving the safety concerns associated with KDs.

## Conclusion

Based on the currently available evidence, KD appears to play a significant role in treating various cardio-metabolic diseases and reveals remarkable effects on cardiovascular function. KD therapy is generally promising in obesity, heart failure, and hypertension, though different voices still exist. In diabetes and dyslipidemia, the performance of KD remains controversial. As for cardiovascular complications of metabolic diseases, current evidence suggests that KD is generally protective to obese related cardiovascular disease (CVD), while remaining contradictory to diabetes and other metabolic disorder related CVDs. Various factors might account for the controversies, including genetic background, duration of therapy, food composition, quality and sources of KDs. Therefore, further studies are warranted to provide concrete and more conclusive opinions. Also, it is vital to monitor safety-related signs and biomarkers during the KD intervention, although most are reversible or transient. In addition, modified KD could be adequately designed and utilized to enhance compliance as a therapeutic approach. Overall, there is a critical need to conduct more rigorous research focusing on the clinical implication and safety issues of KD.

## Author Contributions

All authors listed have made a substantial, direct, and intellectual contribution to the work, and approved it for publication. WYZ: designing, conceptualization, writing, figure plotting, revising; JZ and CDW: designing, funding acquisition, review & editing; XG: funding acquisition, review & editing; LLC: supervision, editing, revising; TC and JYY: figure plotting.

## Funding

The development of this review was supported in whole or in part by grants from the National Natural Science Foundation of China (81770772 to JZ), the Hubei Province Natural Science Foundation (2019CFB701 to JZ), National Natural Science Foundation of China (81803224 to XG) and Young Scholars Program of Shandong University (2018WLJH33 to XG).

## Conflict of Interest

The authors declare that the research was conducted in the absence of any commercial or financial relationships that could be construed as a potential conflict of interest.

## Publisher’s Note

All claims expressed in this article are solely those of the authors and do not necessarily represent those of their affiliated organizations, or those of the publisher, the editors and the reviewers. Any product that may be evaluated in this article, or claim that may be made by its manufacturer, is not guaranteed or endorsed by the publisher.
